# The Economics of Reproducibility in Preclinical Research

**DOI:** 10.1371/journal.pbio.1002165

**Published:** 2015-06-09

**Authors:** Leonard P. Freedman, Iain M. Cockburn, Timothy S. Simcoe

**Affiliations:** 1 Global Biological Standards Institute, Washington, D.C., United States of America; 2 Boston University School of Management, Boston, Massachusetts, United States of America; 3 Council of Economic Advisers, Washington, D.C., United States of America

## Abstract

Low reproducibility rates within life science research undermine cumulative knowledge production and contribute to both delays and costs of therapeutic drug development. An analysis of past studies indicates that the cumulative (total) prevalence of irreproducible preclinical research exceeds 50%, resulting in approximately US$28,000,000,000 (US$28B)/year spent on preclinical research that is not reproducible—in the United States alone. We outline a framework for solutions and a plan for long-term improvements in reproducibility rates that will help to accelerate the discovery of life-saving therapies and cures.

## Abstract 2

This Perspective provides estimates of the rate of irreproducibility of preclinical research and its direct cost implications. It goes on to outline a framework for solutions and a plan for long-term improvements in reproducibility rates.

## Introduction

**Fig 1:**
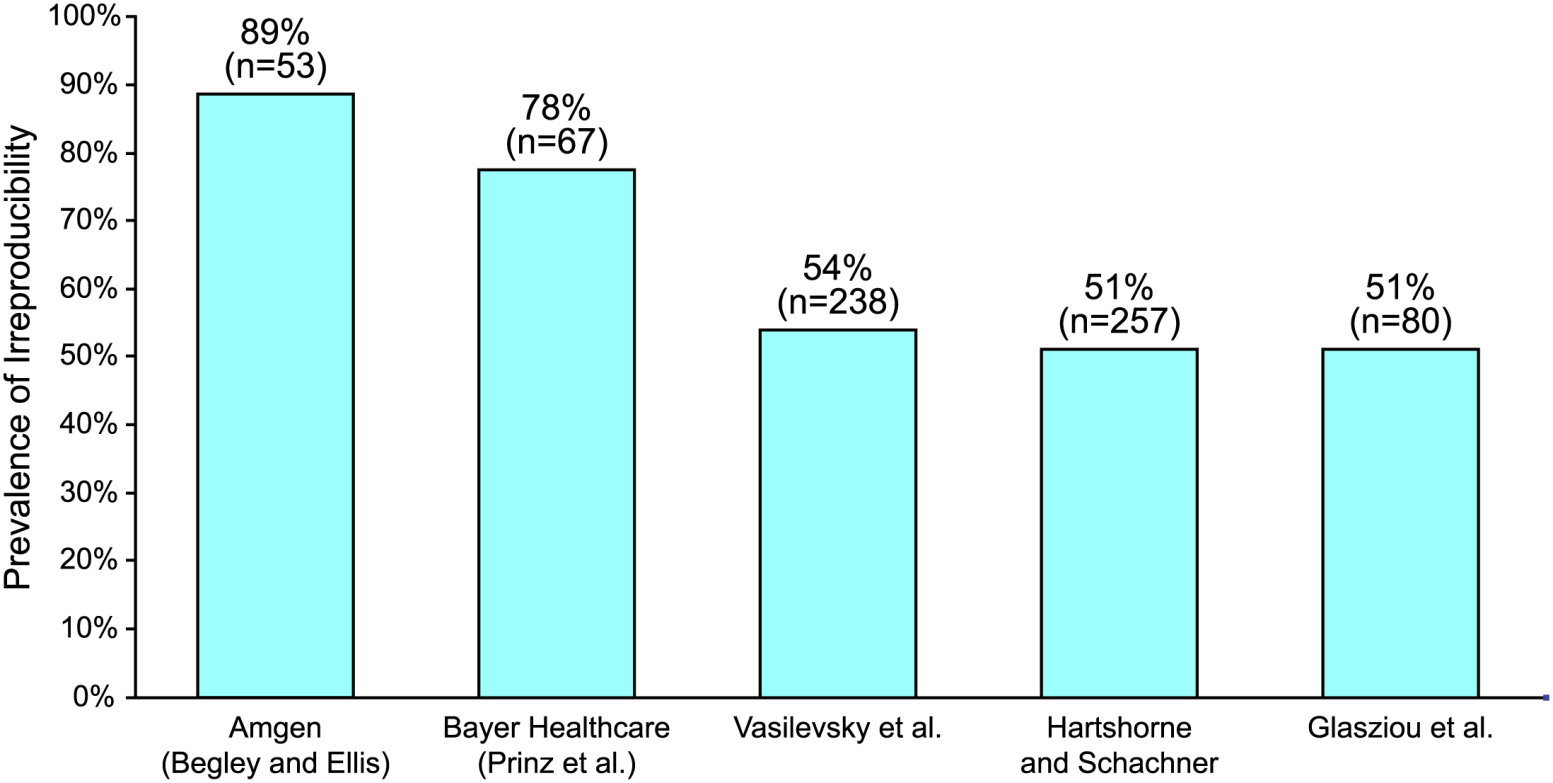
Studies reporting the prevalence of irreproducibility. Source: Begley and Ellis [6], Prinz et al. [7], Vasilevsky [8], Hartshorne and Schachner [5], and Glasziou et al. [9].

Much has been written about the alarming number of preclinical studies that were later found to be irreproducible [1,2]. Flawed preclinical studies create false hope for patients waiting for lifesaving cures; moreover, they point to systemic and costly inefficiencies in the way preclinical studies are designed, conducted, and reported. Because replication and cumulative knowledge production are cornerstones of the scientific process, these widespread accounts are scientifically troubling. Such concerns are further complicated by questions about the effectiveness of the peer review process itself [3], as well as the rapid growth of postpublication peer review (e.g., PubMed Commons, PubPeer), data sharing, and open access publishing that accelerate the identification of irreproducible studies [4]. Indeed, there are many different perspectives on the size of this problem, and published estimates of irreproducibility range from 51% [5] to 89% [6] (Fig 1). Our primary goal here is not to pinpoint the exact irreproducibility rate, but rather to identify root causes of the problem, estimate the direct costs of irreproducible research, and to develop a framework to address the highest priorities. Based on examples from within life sciences, application of economic theory, and reviewing lessons learned from other industries, we conclude that community-developed best practices and standards must play a central role in improving reproducibility going forward.

## Defining Reproducibility

Studies of reproducibility define the phenomenon in a number of ways [10]. For example, some studies define reproducibility as the ability to replicate the same results demonstrated in a particular study using precisely the same methods and materials [11]; others evaluate whether the study’s methodology and results were presented in sufficient detail to allow replication or reanalysis [8]. The definition of reproducibility may also vary depending upon whether a particular study is confirmatory (designed to test basic theories through rigorous study design and analysis) or exploratory (primarily aimed at developing theories and frameworks for further study) [12]. For this paper, we adopt an inclusive definition of irreproducibility that encompasses the existence and propagation of one or more errors, flaws, inadequacies, or omissions (collectively referred to as errors) that prevent replication of results. Clearly, perfect reproducibility across all preclinical research is neither possible nor desirable. Attempting to achieve total reproducibility would dramatically increase the cost of such studies and radically curb their volume. Our assumption that current irreproducibility rates exceed a theoretically (and perhaps indeterminable) optimal level is based on the tremendous gap between the conventional 5% false positive rate (i.e., statistical significance level of 0.05) and the estimates reported below and elsewhere (see S1 Text and Fig 1). Although the optimal statistical power of each study will depend on its objectives, this large gap suggests that published preclinical study results are often less reliable than claimed. From an economic perspective, the system is highly inefficient. While there are several root causes, one overarching source of inefficiency is the continued emphasis on placing responsibility with the researcher—despite the fact that a significant portion of the costs of irreproducibility are ultimately borne by downstream parties in the translation of bench discoveries to bedside therapies [13].

## Analysis of Four Categories of Irreproducibility

**Fig 2:**
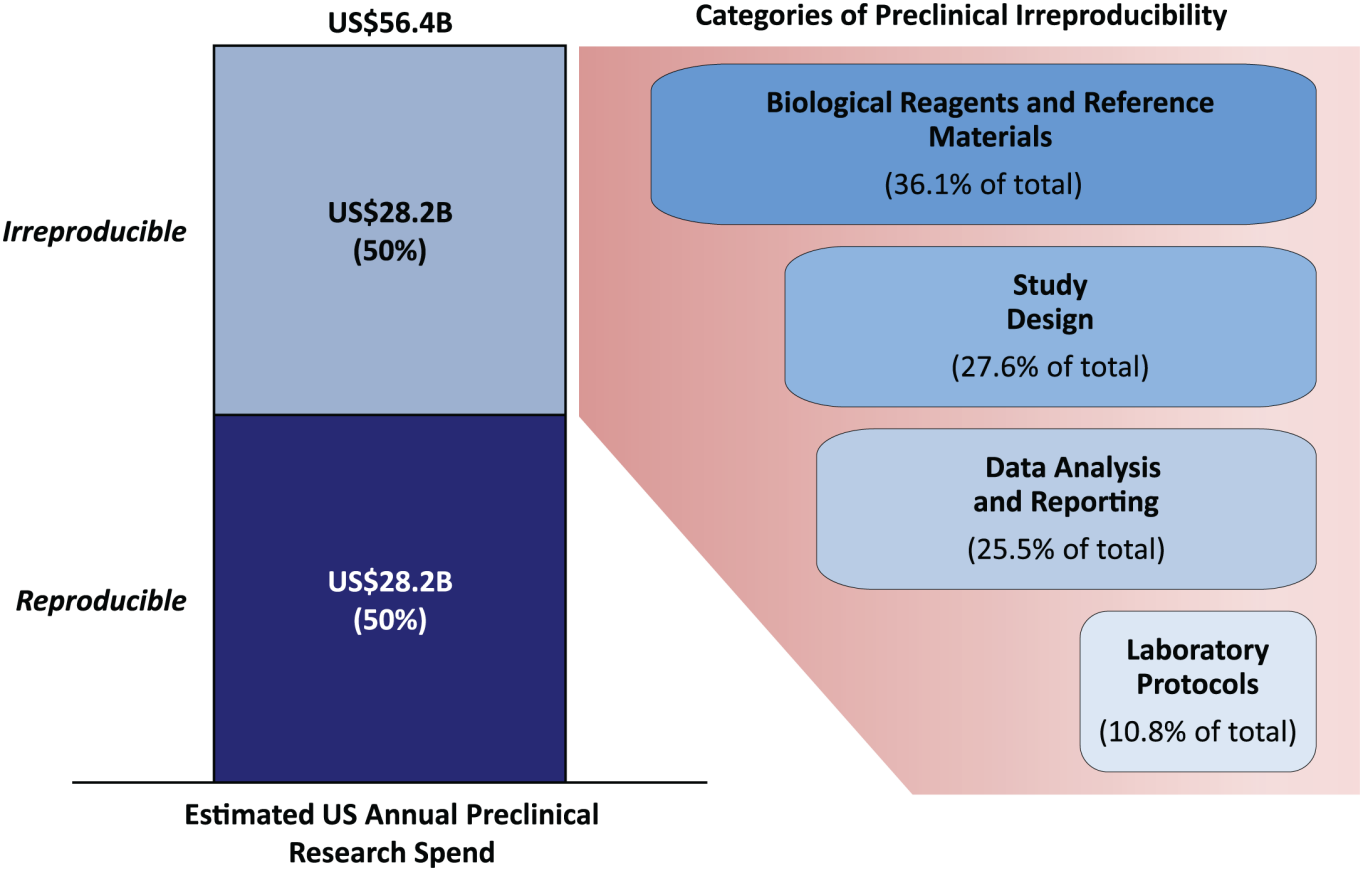
Estimated US preclinical research spend and categories of errors that contribute to irreproducibility. Note that the percentage value of error for each category is the midpoint of the high and low prevalence estimates for that category divided (weighted) by the sum of all midpoint error rates (see S1 Dataset). Source: Chakma et al. [18] and the American Association for the Advancement of Science (AAAS) [19].

Many studies have concluded that the prevalence of irreproducible biomedical research is substantial [1]. The wide range of published estimates reflects the challenges of accurately quantifying and subsequently addressing the problem. Multiple systemic causes contribute to irreproducibility and many can ultimately be traced to an underlying lack of a standards and best practices framework [13]. However, it is reasonable to state that cumulative errors in the following broad categories—as well as underlying biases that could contribute to each problem area [14] or even result in entire studies never being published or reported [15]—are the primary causes of irreproducibility [16]: (1) study design, (2) biological reagents and reference materials, (3) laboratory protocols, and (4) data analysis and reporting. Fig 2, S1 Text, S1 and S2 Datasets show the results of our analysis, which estimates the prevalence (low, high, and midpoint estimates) of errors in each category and builds up to a cumulative (total) irreproducibility rate that exceeds 50%. Using a highly conservative probability bounds approach [17], we estimate that the cumulative rate of preclinical irreproducibility lies between 18% (the maximum of the low estimates, assuming maximum overlap between categories), and 88.5% (the sum of the high estimates, assuming minimal overlap). A natural point estimate of the cumulative irreproducibility rate is the midpoint of the upper and lower bounds, or 53.3%.

### Limitations of the Analysis

This analysis is subject to a number of important limitations, including (1) the small number of studies we were able to identify that provide or support the determination of low, high, and midpoint estimates of prevalence rates for one or more categories of irreproducibility; (2) the lack of consistency as to how reproducibility and irreproducibility are defined across studies; and (3) in some cases, extrapolating from a clinical environment to the preclinical setting when no suitable preclinical studies were available. For these reasons, a rigorous meta-analysis or systematic review was also not feasible. To estimate a theoretically optimal baseline rate of irreproducibility, we would also need data on the financial and opportunity costs of irreproducibility and how these costs (and benefits) vary within the population of preclinical studies. Nonetheless, even simple calculations of direct costs can show that irreproducible preclinical research is a significant problem in terms of lost dollars and lost opportunities for scientific discovery.

## Economic Impact of Irreproducibility

Extrapolating from 2012 data, an estimated US$114.8B in the United States [18] is spent annually on life sciences research, with the pharmaceutical industry being the largest funder at 61.8%, followed by the federal government (31.5%), nonprofits (3.8%), and academia (3.0%) [20]. Of this amount, an estimated US$56.4B (49%) is spent on preclinical research, with government sources providing the majority of funding (roughly US$38B) [19]. Using a conservative cumulative irreproducibility rate of 50% means that approximately US$28B/year is spent on research that cannot be replicated (see Fig 2 and S2 Dataset). Of course, uncertainty remains about the precise magnitude of the direct economic costs—the conservative probability bounds approach reported above suggest that these costs could plausibly be much smaller or much larger than US$28B. Nevertheless, we believe a 50% irreproducibility rate, leading to direct costs of approximately US$28B/year, provides a reasonable starting point for further debate. To be clear, this does not imply that there was no return on that investment. As noted in a recent paper by Stern et al. [21], even in cases of retracted publications due to scientific misconduct, which is not a major source of irreproducibility [13,22], “it is conceivable that some of the research resulting in a retracted article still provides useful information for other nonretracted studies.” However, it does suggest that, even under our relatively conservative assumptions, the impact of the reproducibility problem is economically significant.

Irreproducibility also has downstream impacts in the drug development pipeline. Academic research studies with potential clinical applications are typically replicated within the pharmaceutical industry before clinical studies are begun, with each study replication requiring between 3 and 24 months and between US$500,000 to US$2,000,000 investment [23]. While industry will continue to replicate external studies for their own drug discovery process, a substantially improved preclinical reproducibility rate would derisk or result in an increased hit rate on such investments, both increasing the productivity of life science research and improving the speed and efficiency of the therapeutic drug development processes. The annual value added to the return on investment from taxpayer dollars would be in the billions in the US alone.

## The Role of Best Practices and Standards

Many key stakeholder groups are developing and piloting a range of solutions to help increase reproducibility in preclinical research. For example, the National Institutes of Health (NIH) have recently announced a list of Principles and Guidelines for Reporting Preclinical Research [24], which over 100 journals have joined as cosignatories and that builds on previous recommendations by Landis et al. [25] to improve methodological reporting of animal studies in grant applications and publications. Despite the emergence of a wide variety of reporting guidelines to improve reporting of biomedical research methods and results, to date, compliance levels and their impact to improve reproducibility have been disappointing [26]. Given the size, scale, and complexity of the challenge of reproducibility in preclinical research, there is no single magic bullet solution to the problem. However, one issue that has shown demonstrable impact on similar challenges in other settings is the expanded development and adoption of standards and best practices [13].

In the information and communication technology industries, several standard development organizations have moved beyond simply defining technical interfaces to assume the role of a governing body for critical pieces of shared infrastructure. The Internet is a prime example. The evolution of the Web has been messy; constrained by patent claims, the financial benefit of controlling standards, and confusion over the evolutionary model. However, two organizations, the World Wide Web Consortium (W3C) and the Internet Engineering Task Force (IETF) emerged to develop Web standards and maintain its interoperability as a universal space. The W3C is an excellent example of a successful, internally driven and self-regulating international consortium comprising a public and private partnership working together. Similarly, the IETF operates as a noncommercial/not-for-profit/nongovernmental organization and operates a large number of work groups and informal discussion groups, working on specific, timely issues, then disbanding once these issues are addressed. In the early days of the Internet, both groups successfully steered major global players toward common standards requiring each to compromise and adapt in the short term, but ultimately gain tremendous benefits over the longer horizon.

Although neither example focuses directly on reproducibility, they highlight the importance for the life sciences to engage all stakeholders in a dynamic, collaborative effort to standardize common scientific processes. In the clinical research arena, where the stakes are high and oversight by the US Food and Drug Administration (FDA) is stringent, irreproducibility has been reduced to rates that are generally considered to be scientifically and commercially appropriate [1]. However, this level of stringent oversight often precludes the direct application of clinical methods, practices, and procedures to preclinical research [27]. Furthermore, in a clinical setting, the number of assays and interventions is tightly controlled, which is not typically possible in a basic or preclinical research environment without incurring a significant increase in time and cost. Nonetheless, economic research also has shown that standardization and auditing of biological materials—through biological resource centers—can enhance cumulative production of scientific knowledge by improving both availability and reliability of research inputs [28].

An illustrative example is the use and misuse of cancer cell lines. The history of cell lines used in biomedical research is riddled with misidentification and cross-contamination events [29], which have been estimated to range from 15% to 36% [30]. Yet despite the availability of the short tandem repeat (STR) analysis as an accepted standard to authenticate cell lines, and its relatively low cost (approximately US$200 per assay), only one-third of labs typically test their cell lines for identity [31]. For an NIH-funded academic researcher receiving an average US$450,000, four-year grant, purchasing cell lines from a reputable vendor (or validating their own stock) and then authenticating annually will only cost about US$1,000 or 0.2% of the award. A search of NIH Reporter for projects using “cell line” or “cell culture” suggests that NIH currently funds about US$3.7B annually on research using cell lines. Given that a quarter of these research projects apparently use misidentified or contaminated cell lines, reducing this to even 10% through a broader application of the STR standard—a very realistic goal—would ensure a more effective use of nearly three-quarters of a billion dollars and ultimately speed the progress of research and the development of new treatments for disease.

The economics literature on standardization posits that unless there is a clearly dominant platform leader willing to impose a solution, complex challenges such as irreproducibility that require a coordinated response are best solved by internally organized and driven, dynamic, and self-regulating collaborations of key stakeholders who establish and enforce their respective rules of engagement [32,33]. What is needed is not another list of unfunded mandates, but rather community consensus on priorities for improvement and commitment for the additional funding for implementation. This includes training that focuses specifically on the importance of standards and best practices in basic research in graduate and postdoctoral programs, as well as quality management systems to ensure that best practices are implemented throughout the research process. No doubt that improving training and increasing quality control measures will add costs to the preclinical research enterprise. One estimate in a clinical setting suggests the adoption of mandated quality control procedures would increase costs to 15% to 25% above current spending levels [34]. However, the societal benefits garnered from an increase in reproducible life science research far outweigh the cost. Assuming that we could recover even half of the approximately US$28 billion annually spent on irreproducible preclinical research in the US alone by applying best practices and standards, the savings would be roughly US$14B/year. Moreover, because our analysis indicates that errors in study design and biological reagents and materials contribute to a majority of this spend (see Fig 2), implementing steps to improve preclinical reproducibility should be a priority in these two areas (see Box 1).

**Box 1. Investing in Practical Solutions**

Taking immediate steps in two areas where there will be significant return on investment—study design and biological reagents and reference materials—will yield substantial improvements in preclinical reproducibility rates.

**Study Design**

- Improve training programs at academic institutions to ensure that best practices are reinforced in the areas of core skills, methods, technology, and tools.
- Establish targeted training, coaching, and certification of established principal investigators (PIs) to reinforce application of best practices throughout the research process.
- Establish research funder policies, including funders such as NIH and leading disease foundations, requiring successful completion of training courses at all levels.

**Biological Reagents and Reference Materials**

- Promote broad adoption by vendors to offer only validated reagents (e.g., antibodies and cell lines) and broad utilization of these reagents by PIs as a documented best practice in the research process.
- Ensure that research funder policies require documented use of validated and noncontaminated reagents, annual reagent authentication throughout the research study, and adequate funding to cover these additional costs.
- Ensure that procedures to document reagent validation and lack of contamination are required by publishers.
- Incentivize the continued development of tools for reagent validation using improved genomics data.
- Define standard operating procedures for biological materials handling throughout the material’s lifecycle.

In order to change practices throughout the preclinical research community, all invested stakeholders (academia, journals, industry, and government) must work in partnership to develop, institutionalize, and reward (or even sanction) behaviors, working within a mutually agreed upon set of rules and guiding principles. Such dynamic collaborations could more efficiently represent the needs of all stakeholders and provide unifying guidance and funding suggestions to facilitate meaningful change. Establishing effective collaborative efforts is no simple feat, but we can look to other industries that have been successful in the past as models for the life science community.

## Conclusions

Although differing perspectives on the irreproducibility rate in preclinical research may persist, one fact remains clear: the challenge of increasing reproducibility and addressing the costs associated with the lack of reproducibility in life science research is simply too important and costly to ignore. Lifesaving therapies are being delayed, research budgets face increasing pressure, and drug development and treatment costs are rising. Improving reproducibility remains a critical cornerstone to solving each of these challenges. There are no easy answers to this problem. Real solutions, such as addressing errors in study design and using high quality biological reagents and reference materials, will require time, resources, and collaboration between diverse stakeholders that will be a key precursor to change. Millions of patients are waiting for therapies and cures that must first survive preclinical challenges. Although any effort to improve reproducibility levels will require a measured investment in capital and time, the long-term benefits to society that are derived from increased scientific fidelity will greatly exceed the upfront costs.

## Supporting Information

S1 Text Analysis to determine irreproducibility rate of preclinical research. (DOCX)

S1 Dataset Analysis. (XLSX)

S2 Dataset Economic impact. (XLSX)

## Abbreviations

AAAS: American Association for the Advancement of Science
FDA: US Food and Drug Association
GBSI: Global Biological Standards Institute
IETF: Internet Engineering Task Force
NIH: National Institutes of Health
PI: principal investigator
STR: short tandem repeat
W3C: World Wide Web Consortium
