# Correction: The Economics of Reproducibility in Preclinical Research

**DOI:** 10.1371/journal.pbio.1002626

**Published:** 2018-04-10

**Authors:** The PLOS Biology Staff

There is an error in reference 5. When the manuscript was typeset, the wrong URL was used for the ‘View Article’ link for reference 5. The publisher apologizes for the error. The correct URL for this reference is: https://www.frontiersin.org/articles/10.3389/fncom.2012.00008/full.
