# Supplementary material for: The Economics of Reproducibility in Preclinical Research
Source: PLoS Biol. 2015 Jun 9;13(6):e1002165. doi: 10.1371/journal.pbio.1002165 (PMC4461318; doi:10.1371/journal.pbio.1002165)
Supplement: S1 Text — (DOCX) [file pbio.1002165.s001.docx]

**S1 Text**

**Analysis to Determine the Irreproducibility Rate of Preclinical Research**

The wide range of published estimates of irreproducibility highlights the challenges of accurately quantifying, and subsequently addressing, the problem. To develop our estimate of the current reproducibility rate for preclinical research, we reviewed publicly available data from government sources, industry and analyst reports, and scientific articles. After reviewing and synthesizing all of this information, we then grouped the findings into the following four broad (but not discrete) categories per Freedman and Inglese [[1](#_ENREF_1)], and that align with the chronological stages of study development: (1) study design, (2) biological resources and reference materials, (3) laboratory protocols, and (4) data analysis and reporting.

Our analysis estimates the prevalence of flaws or errors in each category to conservatively build up a cumulative (total) irreproducibility rate for preclinical research that also corresponds with the typical sequence of study development. It is subject to a number of limitations; for example, the small number of identified studies that provide or support the determination of prevalence rates for one or more categories of irreproducibility. Recognizing these limitations, we present ranges of prevalence values (low, high, and midpoint estimates) and relative weightings of contributing factors that support an overall quantification of irreproducibility in preclinical research.

**Four Categories of Irreproducibility**

*Study Design*
 Lack of proper study methodology has been identified as an ongoing challenge to research fidelity for more than 50 years. Improper study design encompasses both studies that are underpowered to yield statistically significant results, as well as those whose design lacks sufficiently rigorous statistical analysis [[2](#_ENREF_2)]. For example, an analysis of 271 animal studies by Kilkenny et al. concluded that 13% used inappropriate statistical methods and almost 60% had problems with both the statistical analysis and the transparency of reporting [[3](#_ENREF_3)]. In addition, the lack of rigor of the experimental design, and in particular the absence of properly blinded studies in confirmatory research, have been cited as key characteristics and contributors to studies and that ultimately are not reproducible [[4](#_ENREF_4)].

For our analysis, we determined an estimate for this category by evaluating research irreproducibility that can be attributed to the routine use of widely accepted statistical testing procedures. Jager and Leek’s [[5](#_ENREF_5)] analysis of *p*-values from more than 75,000 papers from the medical literature concluded a rate of false discoveries among reported results at 14%. Valen Johnson’s[[6](#_ENREF_6)] mathematical calculation of the false results, based on the assumption that 50% of tested null hypothesis are actually true, calculates a false result between 17% and 25% of the time. Using the high and low figures from these studies, we estimate that between 14% and 25% of reported results may be false positives, and used a midpoint of 19.5% for this category.

*Biological Reagents and Reference Materials*
 Reference material flaws are associated with the unreliable identification of source materials used in the preclinical study, particularly contaminated, mishandled, or mislabeled biological reagents like antibodies [[7](#_ENREF_7)] or cell lines [[8](#_ENREF_8)]. A poster child for misidentified cell lines is the adriomycin-resistant breast adenocarcinoma cell lines, MCF-7/AdrR, used in over 300 studies used before they were found to be derived from human ovarian carcinoma cells (now re-designated NCI/ADR-RES) [[9](#_ENREF_9)]. For perspective, based on the cost of an average NIH-funded breast cancer grant (US$370k) in 2013 (<http://report.nih.gov/categorical_spending.aspx>) as much as US$100M of research funding may have been spent using this misidentified cell line alone. Similarly, a recent assessment of mycoplasma contamination found in the NCBI Sequence Read Archive (SRA; <http://www.ncbi.nlm.nih.gov/sra>) conservatively found that 11% of projects were contaminated [[10](#_ENREF_10)], and estimated that hundreds of millions of dollars in NIH-funded research has been potentially affected by widespread mycoplasma contamination of continuous cell lines.

Hughes et al. examined the prevalence of contaminated cancer cell line usage over a period of more than 20 years, and reported a wide range of contamination and mischaracterization, with only a small improvement in rates over time [[11](#_ENREF_11)]. Excluding studies within the Hughes analysis that were outside of the US or had a sample size of <200 cell lines, the range of the reported misidentification or contamination ranged from a low of 14.9% [[12](#_ENREF_12)] to a high of 36% [[13](#_ENREF_13)] (midpoint 25.5%), which serves as our estimated error rate for this category.

*Laboratory Protocols*

Laboratory protocol issues encompass irreproducibility that arises during the preparation and execution of the experiment. Although no study estimating the prevalence of the error rate within preclinical laboratory protocol was identified, analysis within the clinical environment has shown laboratory error rates in the range of 0.3% to 0.5% [[14](#_ENREF_14),[15](#_ENREF_15)]. The error rate within preclinical environment—where there is less use of controls, blinding, and broadly accepted standards and best practices—was assumed to be significantly higher than in clinical trials [[16](#_ENREF_16)]. To estimate the extent to which this assumption holds, we evaluated one study that compared the error rates of other factors in the preclinical to clinical environment, where estimates of the error rates in the preclinical laboratory were found to be as high as 19 times the clinical rate [[17](#_ENREF_17)]. Applying this multiplier against the error rates for the preclinical laboratory setting generates an estimate of 5.7% to 9.5%, with a midpoint of 7.6%.

*Data Analysis and Reporting*

The fourth category of contributing factors to preclinical irreproducibility is the analysis and reporting of data. Data sharing and reporting has been recognized by the NIH [[18](#_ENREF_18)] as an essential part of the translational research process, and together with the rise of post-publication review [[19](#_ENREF_19)], it is a key factor in facilitating the identification of irreproducible data or studies [[20](#_ENREF_20)]. The same animal study referenced earlier (Kilkenny et al.) also looked at design issues and concluded that only 59% of the papers studied had included satisfactory level of details on the methodology, sample size, and key characteristics of the animals used in the study [[3](#_ENREF_3)]. And while less common, errors in analysis can have devastating impacts, as two researchers reported in 2007 when a simple calculation error (change in sign) undermined several years of work on multidrug resistance efflux transporters and that led to the retraction of a widely cited paper [[21](#_ENREF_21)].

A number of studies have investigated the issue of inadequate reporting of research, with estimates of improper reporting reaching as high as 87% [[22](#_ENREF_22)], although the impact of such data analysis and reporting errors on irreproducible research is inconclusive. For our analysis, we used the results of a study of 234 clinical trials, which found that 18% of data reported was deemed to be “inadequate” [[23](#_ENREF_23)], which provides a conservative, lower-end estimate for the impact of this category on overall irreproducibility.

**Cumulative Irreproducibility Rate**

In order to calculate the total rate of irreproducibility in preclinical research, the estimated prevalence values for all four categories were used as outlined in S1 and S2 Datasets. Given the limited number of studies in which we were able to identify reporting incidence rates for irreproducibility, and a lack of consistency as to how reproducibility/irreproducibility is defined, a rigorous meta-analysis or systematic review was not feasible. However, using both the range and midpoint estimates for each category of error, the combined impact was calculated using a highly conservative *probability bounds* approach [[24](#_ENREF_24)] with the cumulative irreproducibility rate estimated to exceed 50% (see Fig. 2 and S1 Dataset).

**Comparison to Prior Estimates of Irreproducibility**

Several prominent studies have examined the prevalence of irreproducibility within the confines of the research at a specific company or academic institution. One widely discussed effort was Amgen scientists’ ability to replicate only 6 (11%) of 53 key oncological studies [[25](#_ENREF_25)]. A similarly low reproducibility rate was seen at Bayer, whose study concluded that a mere 20 to 25% of published data over a 4-year period could be corroborated internally [[26](#_ENREF_26)]. Likewise, researchers at the Oregon Health & Science University found that 54% of 238 biomedical papers published in 84 journals failed to identify all of the resources necessary to reproduce results [[27](#_ENREF_27)]. And finally, a review of 80 studies published in the journal *Evidence-Based Medicine* found that fewer than half (49%) included sufficient details of results to accurately attempt replication [[28](#_ENREF_28)]. Notably, authors of the latter advocate for tracking replication as a means of post-publication evaluation to both assist researchers to identify reliable findings and to explicitly recognize and incentivize the publication of reproducible data and results. Our calculated estimate (53.3%) of the cumulative prevalence of irreproducible preclinical research falls well within the boundaries of the results published in these previous studies (Fig. 1).

**References**

1. Freedman LP, Inglese J (2014) The increasing urgency for standards in basic biologic research. Cancer Res 74: 1-6.

2. Ioannidis JPA (2005) Why most published research findings are false. PLoS Med 2: e124.

3. Kilkenny C, Parsons N, Kadyszewski E, Festing MF, Cuthill IC, et al. (2009) Survey of the quality of experimental design, statistical analysis and reporting of research using animals. PLoS One 4: e7824.

4. Begley CG (2013) Reproducibility: six red flags for suspect work. Nature 497: 433-434.

5. Jager LR, Leek JT (2014) An estimate of the science-wise false discovery rate and application to the top medical literature. Biostat 15: 1-12.

6. Johnson VE (2013) Revised standards for statistical evidence. PNAS 110: 19313-19317.

7. Bradbury ARM, Plückthun A (2015) Reproducibility: Standardize antibodies used in research. Nature 518: 27-29.

8. Lorsch JR, Collins FS, Lippincott-Schwartz J (2014) Cell Biology. Fixing problems with cell lines. Science 346: 1452-1453.

9. Liscovitch M, Ravid D (2007) A case study in misidentification of cancer cell lines: MCF-7/AdrR cells (re-designated NCI/ADR-RES) are derived from OVCAR-8 human ovarian carcinoma cells. Cancer Lett 245: 350-352.

10. Olarerin-George AO, Hogenesch JB (2014) Assessing the prevalence of mycoplasma contamination in cell culture via a survey of NCBI's RNA-seq archive. bioRxiv: Cold Spring Harbor Laboratory. pp. 1-24.

11. Hughes P, Marshall D, Reid Y, Parkes H, Gelber C (2007) The costs of using unauthenticated, over-passaged cell lines: how much more data do we need? Biotechniques 43: 575, 577-578, 581-572 passim.

12. Drexler HG, Dirks WG, Matsuo Y, MacLeod RA (2003) False leukemia-lymphoma cell lines: an update on over 500 cell lines. Leukemia 17: 416-426.

13. Hukku B, Halton DM, Mally M, Peterson WD, Jr. (1984) Cell characterization by use of multiple genetic markers. Adv Exp Med Biol 172: 13-31.

14. Carraro P, Plebani M (2007) Errors in a stat laboratory: types and frequencies 10 years later. Clin Chem 53: 1338-1342.

15. Wiwanitkit V (2001) Types and frequency of preanalytical mistakes in the first Thai ISO 9002:1994 certified clinical laboratory, a 6 - month monitoring. BMC Clin Pathol 1: 5.

16. Collins FS, Tabak LA (2014) NIH plans to enhance reproducibility. Nature 505: 612-613.

17. Lumbreras B, Parker LA, Porta M, Pollan M, Ioannidis JP, et al. (2009) Overinterpretation of clinical applicability in molecular diagnostic research. Clin Chem 55: 786-794.

18. Roth KA, Cox AE (2015) Science isn't science if it isn't reproducible. Am J Pathol 185: 2-3.

19. Freedman LP, Gibson MC (2015) The impact of preclinical irreproducibility on drug development. Clin Pharmacol Therap 97: 16-18.

20. Christakis DA, Zimmerman FJ (2013) Rethinking reanalysis. JAMA 310: 2499-2500.

21. Ma C, Chang G (2007) Structure of the multidrug resistance efflux transporter EmrE from *Escherichia coli*. Proc Natl Acad Sci U S A 104: 3668.

22. Chalmers I, Glasziou P (2009) Avoidable waste in the production and reporting of research evidence. Lancet 374: 86-89.

23. Hewitt C, Hahn S, Torgerson DJ, Watson J, Bland JM (2005) Adequacy and reporting of allocation concealment: review of recent trials published in four general medical journals. BMJ 330: 1057-1058.

24. Manski CF (2003) Partial Identification of Probability Distributions. In: Manski CF, editor. Springer Series in Statistics New York, New York, USA: Springer International Publishing AG. pp. 178.

25. Begley CG, Ellis LM (2012) Drug development: raise standards for preclinical cancer research. Nature 483: 531-533.

26. Prinz F, Schlange T, Asadullah K (2011) Believe it or not: how much can we rely on published data on potential drug targets? Nat Rev Drug Discov 10: 712-712.

27. Vasilevsky NA, Brush MH, Paddock H, Ponting L, Tripathy SJ, et al. (2013) On the reproducibility of science: unique identification of research resources in the biomedical literature. PeerJ 1: e148.

28. Hartshorne JK, Schachner A (2012) Tracking replicability as a method of post-publication open evaluation. Front Comput Neurosci 6: 1-13.
